# Photoactive Protochlorophyllide-Enzyme Complexes Reconstituted with PORA, PORB and PORC Proteins of *A. thaliana*: Fluorescence and Catalytic Properties

**DOI:** 10.1371/journal.pone.0116990

**Published:** 2015-02-06

**Authors:** Michał Gabruk, Anna Stecka, Wojciech Strzałka, Jerzy Kruk, Kazimierz Strzałka, Beata Mysliwa-Kurdziel

**Affiliations:** 1 Department of Plant Physiology and Biochemistry, Faculty of Biochemistry, Biophysics and Biotechnology, Jagiellonian University, Krakow, Poland; 2 Department of Plant Biotechnology, Faculty of Biochemistry, Biophysics and Biotechnology, Jagiellonian University, Krakow, Poland; National Taiwan University, TAIWAN

## Abstract

Photoactive Pchlide-POR-NADPH complexes were reconstituted using protochlorophyllide (Pchlide) and recombinant light-dependent protochlorophyllide oxidoreductase (POR) proteins, His₆-PORA, His₆-PORB and His₆-PORC, from *Arabidopsis thaliana*. We did not observe any differences in the kinetics of the protochlorophyllide photoreduction at room temperature among the PORA, PORB and PORC proteins. In contrast, the PORC protein showed lower yield of Chlide formation than PORA and PORB when preincubated in the dark for 30 min and then illuminated for a short time. The most significant observation was that reconstituted Pchlide-POR-NADPH complexes showed fluorescence maxima at 77 K similar to those observed for highly aggregated Pchlide-POR-NADPH complexes in prolamellar bodies (PLBs) *in vivo*. Homology models of PORA, PORB and PORC of *Arabidopsis thaliana* were developed to compare predicted structures of POR isoforms. There were only slight structural differences, mainly in the organisation of helices and loops, but not in the shape of whole molecules. This is the first comparative analysis of all POR isoforms functioning at different stages of *A. thaliana* development.

## Introduction

Light-dependent protochlorophyllide oxidoreductase (POR, EC.1.3.1.33) is one of the two enzymes in nature that catalyse the reduction of protochlorophyllide (Pchlide). It operates in all photosynthetic organisms except anoxygenic photosynthetic bacteria (for a review see [1–2]). In angiosperms, POR is the only enzyme capable of the catalysis of Pchlide reduction. POR is a single polypeptide enzyme (~ 36 kDa) that shows a high degree of sequence homology among different organisms [3]. It is a member of the superfamily of SDR proteins, *i*.*e*. Short-chain Dehydrogenases/Reductases [3] (for a review see [2]). Some structural features, like the characteristic pattern of α/β folding, the nucleotide binding site and the catalytic motif are characteristic of this superfamily (see [4]). The unique feature of POR is light-triggered catalytic activity, so that this enzyme is also regarded as a photoenzyme.

Pchlide is one of last intermediates of chlorophyll (Chl) biosynthesis (for a review see [1], [5]). It is a porphyrin that has a Mg^2+^ ion coordinated in a tetrapyrrole ring. The conversion of Pchlide to Chl, which is the main photosynthetic pigment, involves two reactions: (1) the reduction of the C17 = C18 double-bond in the porphyrin ring, leading to chlorophyllide (Chlide) formation, and (2) the esterification of Chlide by phytol or its unsaturated precursors [6]. The reduction of Pchlide in angiosperms, which is catalysed by POR and thus light-triggered, plays a regulatory role both in Chl biosynthesis (*e*.*g*. reviewed in [1], [5], [7]) and in plant development (*e*.*g*. reviewed in [7–8]).

In dark-grown seedlings, Pchlide accumulates and forms complexes with POR and NADPH. These ternary complexes have been detected in a highly regular lipid structure known as the prolamellar body (PLB), characteristic of etioplasts (for more details see [7] and references therein). The spectral properties of Pchlide *in vivo* and in isolated membranes have been intensively investigated using absorption and fluorescence spectroscopy at 77 K, and several Pchlide forms have been described (for a review see: [8–10]). Nevertheless, the organisation of Pchlide-POR-NADPH complexes *in vivo* is still a matter of debate. Further systematic investigations are needed to solve this problem and understand the molecular interactions within photoactive Pchlide-POR-NADPH complexes as well as the interactions of these complexes with lipid membranes.

In angiosperms, two POR isoforms, *i*.*e*. PORA and PORB, were first identified in *Hordeum vulgare* [11] and in *Arabidopsis thaliana* [12], and then in other plant species. PORA transcripts accumulate in young etiolated seedlings and undergo strong down-regulation by light, whereas PORB transcripts, which were also detected in dark-grown seedlings, remain detectable at later stages of development and also in the light. The role of PORA and PORB in PLB formation is still under debate (see [7] for discussion). The third protein, PORC, has only been found in *A*. *thaliana* so far [13–14]. PORC transcripts were detected in response to light in fully matured green tissues, as well as in greening ones. Different light-regulation of POR proteins of Arabidopsis indicate that plants may use preferentially one of the three enzymes under a given light regime to keep the optimal level of Chl synthesis [14]. *A*. *thaliana* is the only plant so far recognised where three different POR isozymes participate in the regulation of Chl biosynthesis [15] (reviewed by Masuda [1]), however, the functional assembly and activity of these enzymes has not been characterised *in vitro* and *in vivo* in detail.

In the present paper we obtained recombinant PORA, PORB and PORC from *Arabidopsis thaliana* and used them for reconstitution and fluorescence characteristics of photoactive substrate—enzyme complexes. We built homology models of *A*. *thaliana* POR isozymes to reveal to what extent certain sequence differences among them might affect the protein structure. Because POR crystallisation has not yet been achieved, homology modelling is the only way to carry out such an analysis.

## Material and Methods

### Construction of expression vectors

cDNA coding for *Arabidopsis thaliana* PORA, PORB and PORC were purchased from Arabidopsis Biological Resource Centre. The POR open reading frames were amplified using PCR under the following conditions: 95°C for 2 min, 25 cycles of 95°C for 30 sec, 60°C for 30 sec, 72°C for 2 min, with a final extension of 72°C for 7 min. The 50 μl of the reaction mixture consisted of 1 × PCR buffer (20 mM Tris-HCl, pH 8.5; 10 mM KCl; 10 mM (NH_4_)_2_SO_4_; 2 mM MgSO_4_; 0.1% Triton X-100), 0.2 mM dNTP, 2 μM of appropriate primer set (PORAF: 5’-GAGCATATGGCAATCGCGACTTCAACTCCATC-3‘, PORAR: 5‘-AGAGCTCGAGTTAGGCCAAGCCTACGAGCTTC-3; PORBF: 5’-ATACATATGACCGCTGCGACTTCAAGCCCT-3‘, PORBR: 5‘-ATTCTCGAGTTAGGCCAAGCCCACGAG-3‘; PORCF: 5‘-ATACATATGACAGTTACAGCCACGCCGCCGGCA-3‘, PORCR: 5‘-AAGGGATCCTCATGCCAAACCAACAAGCTTC-3‘), 10 ng of DNA template and a 1 U of WALK polymerase (A&A Biotechnology, Poland). Determination of the cleavage site between the transit peptide and the mature POR was performed with respect to the recently published data [16] and the possibility of obtaining soluble recombinant POR proteins. The PCR products were purified form agarose gel with the help of a Gel-Out DNA purification kit (A&A Biotechnology, Poland). Next, a pET15b vector (Novagen, USA) and the purified PCR products were digested using NdeI and XhoI (ThermoScientific, USA) restriction enzymes and purified using a Clean-up DNA purification kit (A&A Biotechnology, Poland). Finally, the inserts were ligated into the prepared pET15b vector and the ligation mixtures were transformed into DH5α competent cells. The plasmid containing cells were selected on an agar medium supplemented with 100 mg/l of ampicillin. The inserts containing clones were identified using colony PCR. The recombinant plasmids were isolated using plasmid purification kit (A&A Biotechnology, Poland) and the inserts were verified by sequencing (Genomed, Poland).

### POR expression and purification

*E*. *coli* BL21(DE3)pRILcells containing the expression construct were grown in an LB medium supplemented with 100 mg/l ampicillin and 25 mg/l chloramphenicol at 37°C with rotary shaking. When the OD_600_ of the culture was 0.7 the protein overexpression was induced with 0.25 mM isopropyl-D-thiogalactoside (IPTG) for 2 h at 22°C. The culture was then centrifuged at 15 000 × *g* and at 4°C for 4 min and a bacterial pellet was frozen at—20°C for further purification. The bacterial pellet was suspended in a WEB buffer (50 mM Na_2_HPO_4_/NaH_2_PO_4_ buffer, pH 7.0, with 7 mM β-mercaptoethanol) containing 1 mM phenylmethanesulfonefluoride (PMSF). Cells were lysed by sonication using a Sonics Vibra cell VC-505 sonicator. The sonication was performed for 6 minutes (5 sec pulse/ 15 sec break cycles) on ice. Then the lysate was centrifuged at 14 000 × *g* and at 4°C for 1 h. The supernatant was incubated with 5 ml of TALON metal affinity resin (Clontech, USA) at 4°C for 2 h with gentle rotary agitation. The resin was centrifuged (700 × *g* and at 4°C for 5 min) and washed with the WEB buffer. The protein was eluted using WEB buffer supplemented with 200 mM imidazole, supplied with glycerol to a final concentration of 25% and frozen at -20°C for further analysis. POR concentration was estimated using the Bradford reagent (Sigma Aldrich, UK).

### Protochlorophyllide isolation

Wheat (*Triticum aestivum L*.) plants were grown hydroponically in darkness at 25°C for 6 days on a modified Hoagland medium (see [17] for details). Leaves were cut, treated overnight with δ-aminolevulinic acid (ALA) and used for Pchlide extraction with acetone. Pchlide (*i*.*e*. MV-Pchlide *a*) was then separated from other pigments and purified using HPLC. The protocol of Pchlide isolation and purification was already published by Kruk and Mysliwa-Kurdziel [17]. The organic solvents used for Pchlide purification were of analytical or HPLC grade.

### Preparation of Pchlide-POR-NADPH complexes for studies at 77 K

Photoactive Pchlide-POR-NADPH complexes were reconstituted in a 37 mM sodium phosphate buffer (Na_2_HPO_4_/NaH_2_PO_4_), pH 8.3 containing 150 mM imidazole, 5 mM β-mercaptoethanol, 25% glycerol and 0.2 mM NADPH. A small portion of the stock solution of POR, which was stored at—20°C, was gently thawed on ice just before the experiments. First, an aliquot of POR, having concentration between 4 and 11 μM, was added to the reconstituting buffer at ambient temperature and gently mixed. Then, a suitable aliquot of Pchlide in methanol (268 μM) was added to the mixture to obtain desired Pchlide:POR ratio and gently mixed. The sample was incubated for 30 min at room temperature, then placed in quartz capillaries and frozen in liquid nitrogen for fluorescence measurement at—196 C (*i*.*e*. 77 K). The total volume of the reconstituting mixture in a single experiment was 100 μl where the content of methanol did not exceed 2%. In some experiments, especially for low Pchlide:POR ratios the Pchlide stock solution (268 μM) was diluted with methanol by 2-, 5- or 10 times. All the manipulations were performed under dim and scattered green light. This light source was previously shown not to induce Pchlide photoreduction in etiolated leaves.

After taking the fluorescence spectrum of the reaction mixture in the dark, each sample was tested for the photoactivity of the reconstituted Pchlide-POR-NADPH complexes. Capillaries with samples were quickly thawed in the dark to the ambient temperature and illuminated for 15 s with white light (8 μmol m^-2^ s^-1^ photon flux density). Then they were frozen again in liquid nitrogen for the measurement of the fluorescence emission spectra to observe Pchlide photoreduction.

For each POR protein, at least two series of experiments were performed for at least six different Pchlide:POR ratios.

### Fluorescence measurements

A Perkin Elmer LS-50B spectrofluorometer equipped with sample stirring was used for steady-state measurements of fluorescence spectra at room temperature. Low temperature (*i*.*e*. at 77 K) emission and excitation spectra were measured using a special device with a quartz capillary of 3 mm diameter as the sample holder, which was cooled with liquid nitrogen. Fluorescence emission spectra were usually recorded in the range from 600 (or 605 nm) to 750 nm with the scanning speed of 120 nm/min. The data collection frequency was 0.5 nm. The excitation wavelength was 440 nm. Fluorescence excitation spectra were measured for given emission wavelengths (see results for details) in the range between 400 and 500 nm. Excitation and emission slits were 10 nm.

### POR kinetics at room temperature

POR enzymatic activity was examined at ambient temperature in a 37.5 mM sodium phosphate buffer (Na_2_HPO_4_/NaH_2_PO_4_), pH 8.3, containing 5mM β-mercaptoethanol, 150 mM imidazole, 0.05 mM NADPH and glycerol (25%, v/v). The reaction was performed in a fluorescence cuvette (*i*.*e*. 1×1 cm cuvette, for a sample volume of 2 ml) placed in the fluorometer’s sample holder (Perkin Elmer LS-50B). Pchlide photoreduction was induced by the spectrofluorometer lamp (0.45 μmol m^-2^ s^-1^ photon flux density). The first fluorescence emission spectrum was measured immediately after the addition of Pchlide to a reaction mixture containing POR and NADPH. Then at least fifteen spectra were automatically collected one after another for several minutes, with stirring of the sample. The sample was constantly under illumination. To shorten the time of a single spectrum recording, the scanning speed was increased to 240 nm/min. The excitation wavelength was 440 nm. For each POR protein, a series of spectra were measured for several freshly prepared samples with different Pchlide concentrations, to obtain a given range of Pchlide:POR ratio. POR concentration was within the range of 1–2.75 μM in these measurements.

### Homology models of POR isoforms

**Fig 1:**
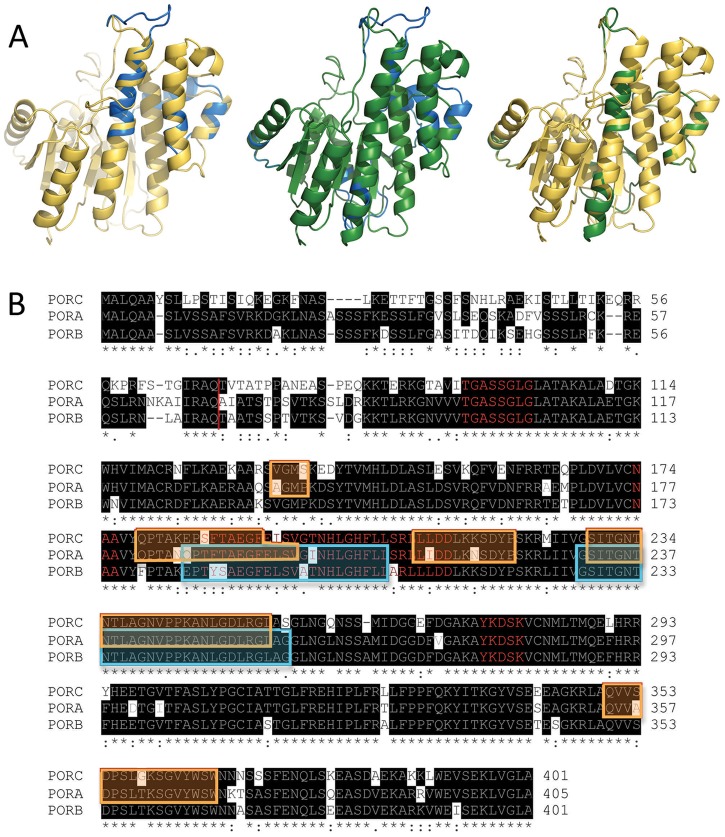
Homological models and amino acid sequences of POR proteins. (A) Aligned homological models of PORA (blue), PORB (yellow) and PORC (green). Structures of pairs of proteins (PORA/PORB, PORA/PORC and PORB/PORC) are overlaid. (B) Aligned sequences of PORC, PORA and PORB. Identical residues are highlighted in black. Red line marks transit peptide cleavage site. Some mismatches in the structure alignments obtained between PORA and PORC are marked in the orange frame, while those between PORA and PORB—in the cyan one. Characteristic motifs are marked with red letters, namely the G-rich motif, the NAA motif, the TFT motif and the catalytic YxxxK motif.

Homology modelling was performed using the Phyre2 algorithm [18] for PORA, PORB and PORC sequences lacking a transit peptide (see Fig. 1B). The best suitable template for modelling purposes was porcine testicular carbonyl reductase (PDB id: 1n5d) providing 81% sequence coverage with 100% confidence. Sequence and structure alignment was conducted with ClustalW [19] and PyMOL (The PyMOL Molecular Graphics System, Version 1.5.0.4 Schrödinger, LLC), respectively.

## Results

### Homology modelling of Arabidopsis PORA, PORB and PORC

The structures of the *A*. *thaliana* PORA, PORB and PORC proteins obtained as a result of modelling are shown in Fig. 1A. The structures are similar for all the proteins and exhibit characteristic features of SDR proteins [4], which is to say that they consist of two long helices facing 7-stranded beta sheets and a few shorter helices arranged in the shape of an oblate spheroid.

As expected, there was a highly similar sequence alignment (73% identical residues) among POR isoforms (Fig. 1B). The characteristic and conservative motifs for SDR proteins [4] are present among all the isoforms, *i*.*e*. the catalytic motif (*YxxxK*) and the nucleotide binding motifs (the G-rich motif and the *NAA* motif). The lowest homology was found at the N-terminal end of the protein, *i*.*e*. up to 83–79 amino acid residues (*aa*), although a large part of this region is within the transit peptide, which is absent in the mature enzyme and thus does not affect the enzymatic properties of proteins.

On the basis of the analysis of pairs of overlaid POR isozymes, some minor differences in helixes and loop arrangement may be observed, which are marked both in sequence and structure alignments (Fig. 1). Some differences were found between 232 *aa* and 256 *aa* (the numeration for PORA), which correspond to “the extra loop” described in the first published model of *Synechocystis* POR [20]. In addition, some mismatches between PORA and C were noticed between 182 *aa* and 197 *aa* (the numeration for PORA), which is located in the N-terminal end of the TFT motif, which was recently described by our group [21]. Moreover, PORC structure also differs from PORA at the C-terminal end of the TFT motif and in two other fragments of unknown function (see Fig. 1B). Nevertheless, the detailed analysis of differences among the obtained structures (marked as coloured frames in Fig. 1B) and *aa* sequence showed that differences in modelled structure do not match dissimilarities in the sequences.

### Low temperature fluorescence of Pchlide-POR-NADPH complexes

**Fig 2:**
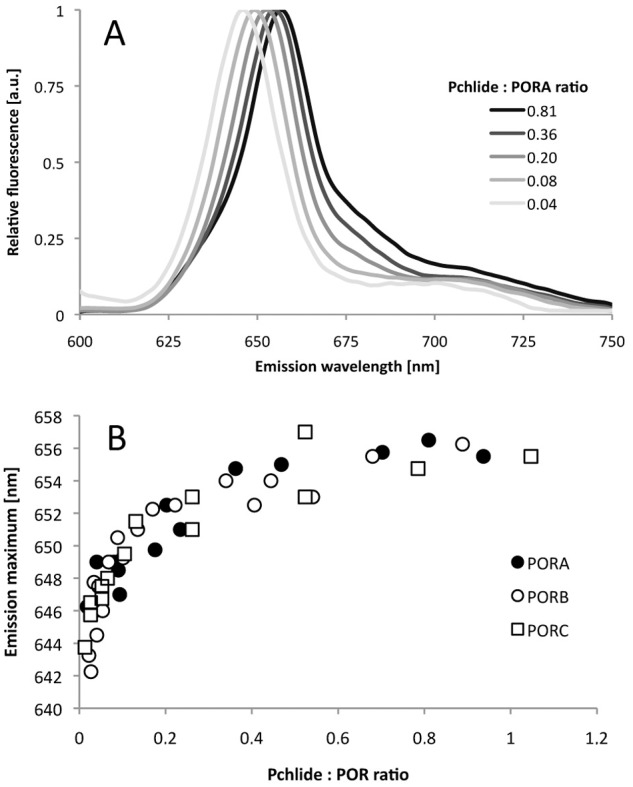
Fluorescence studies of reaction mixtures containing PORA, Pchlide, and NADPH (A) Representative 77 K fluorescence emission spectra; the Pchlide:PORA ratio is shown in the legend. Excitation wavelength: 440 nm. (B) The relation between the position of the 77 K fluorescence emission maximum of Pchlide (determined on the basis of spectra similar to those shown in Fig. 2A) and the Pchlide:POR ratio in reaction mixtures containing Pchlide, NADPH and either PORA, PORB or PORC, respectively. The presented results were obtained from several experiments performed for different POR concentration between 4 and 11 μM, and therefore were analysed with respect to Pchlide:POR ratio.

Fluorescence spectroscopy at 77 K is a convenient tool for analysing Pchlide properties in reconstituted substrate-enzyme complexes and for verifying the ability of the recombinant POR enzyme to catalyse Pchlide photoconversion. In our study, Pchlide was added in the dark to a buffer solution containing POR and NADPH (0.20 mM), then incubated for 30 min and frozen in liquid nitrogen. Representative emission spectra of the samples reconstituted with PORA are shown in Fig. 2A, whereas results of control experiments performed for different reaction assay compositions are shown in S1 Fig. Depending on the Pchlide:POR ratio, the maximum varied between 642 and 657 nm. In the case of a high Pchlide:POR ratio (*i*.*e*. higher than 1) the main fluorescence band was accompanied by a new fluorescence band at shorter wavelengths (not shown), which made the analysis much complicated. Therefore, in the present paper, we presented only results obtained for the Pchlide:POR ratio lower than one.

The dependence between the position of the fluorescence emission maximum at 77 K and the Pchlide:POR ratio in the reaction mixtures prepared for PORA, PORB and PORC, respectively, is shown in Fig. 2B. For very low Pchlide:POR ratios, *i*.*e*. lower than 0.1, a strong red-shift of the maximum was observed with increasing relative Pchlide content, which became less pronounced for high Pchlide:POR ratios. Finally, the fluorescence maximum was observed at 655.4 ± 0.6 nm without any differences among POR isoforms and irrespectively of further increase in the Pchlide:POR ratios.

**Fig 3:**
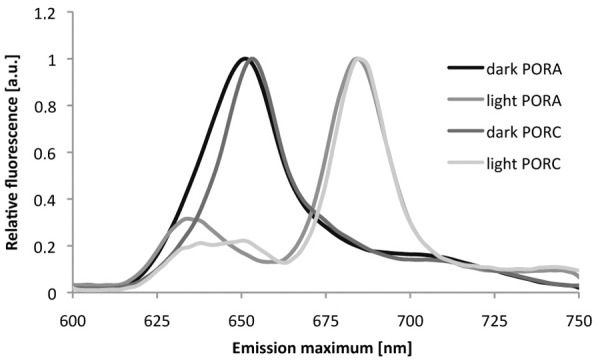
Representative 77 K fluorescence emission spectra of a reaction mixture containing Pchlide, NADPH and PORA or PORC. Spectra labelled as “dark” were measured after a 30-min incubation of the reaction mixture in darkness. After these measurements, the samples were thawed, illuminated, frozen again and used for fluorescence measurement (spectra labelled as “light”). See materials and methods for the details. POR concentration: 6.3 ± 0.3 μM, Pchlide concentration: 1.3 μM, NADPH concentration: 0.2 mM. Pchlide:POR ratio = 0.21. Excitation wavelength: 440 nm.

The reconstituted Pchlide-POR-NADPH complexes were photoactive, *i*.*e*. Pchlide was converted to Chlide upon a short illumination at room temperature of samples prepared in the dark. The photoconversion effect was demonstrated with fluorescence spectra measured at 77 K. The representative spectra measured for Pchlide:POR ratio of 0.2 are shown in Fig. 3. NADPH was required for the photoactivity (see control samples in S1 Fig.). The intensity of the Pchlide fluorescence band decreased and a new band of Chlide fluorescence, having a maximum between 677 and 689 nm, appeared. The illumination clearly revealed the existence of a pool of Pchlide that remains not reduced and which shows a fluorescence maximum at around 640 nm. Both the shape and the position of this band differed among POR isozymes. In the case of PORA and PORB, it was a single band having a maximum at 636 ± 3 nm, whereas for PORC, it was composed of two peaks at 636 and 650 nm (Fig. 3). The latter, however, disappeared after 1 minute of illumination (S2 Fig.).

**Fig 4:**
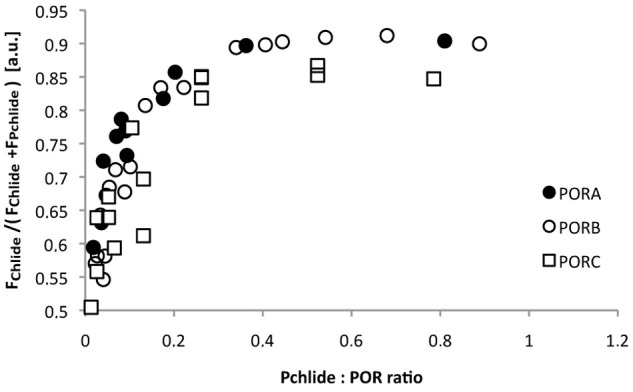
The dependence of the relative Chlide fluorescence intensity on the Pchlide:POR ratio. The relative Chlide fluorescence intensity was calculated as F_Chlide_/(F_Chlide_+F_Pchlide_). Fluorescence intensity of Chlide (F_Chlide_) and Pchlide (F_Pchlide_) were read from 77 K fluorescence emission spectra, around 680 and 640 nm, respectively, that were measured for reaction mixtures after 15 sec of illumination with white light (8 μmol m^-2^ s^-1^ photon flux density). Excitation wavelength: 440 nm. The presented results were obtained from several experiments performed for different POR concentration between 4 and 11 μM, and therefore were analysed with respect to Pchlide:POR ratio.

The fluorescence spectra of the reaction mixture measured at 77 K after 15 s illumination were also used to estimate the extent of Chlide formation. The relative Chlide fluorescence, *i*.*e*. the ratio of the maximal Chlide fluorescence intensity to the sum of the maximal intensities of Chlide and Pchlide fluorescence bands, was calculated for each spectrum (Fig. 4). The extent of Chlide formation increased with an increasing Pchlide:POR ratio, especially for low pigment:enzyme proportions. It remained slightly lower for PORC than for PORA and PORB, especially for high Pchlide:POR ratios.

**Fig 5:**
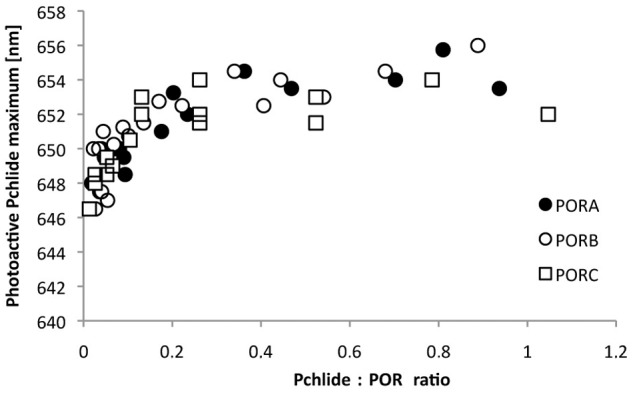
The dependence of the maximum of the photoactive Pchlide-POR-NADPH complexes on the Pchlide:POR ratio. Maxima were read from the “dark”-“light” difference spectra obtained for substraction of similar spectra to those shown in Fig. 3 but measured for all the investigated Pchlide:POR ratios. The presented results were obtained from several experiments performed for different POR concentration between 4 and 11 μM, and therefore were analysed with respect to Pchlide:POR ratio.

The observation of only partial Pchlide photoreduction under these conditions requires the determination of the exact position of the fluorescence maximum of Pchlide bound in photoactive substrate-enzyme complexes. This was achieved by the calculation of dark minus light difference spectra (“dark-light”), for the spectra which were normalised at 622.5 nm. Fluorescence maxima of Pchlide bound to Pchlide-POR-NADPH complexes were observed between 646 and 656 nm and no clear differences were observed among the different POR proteins (Fig. 5). A red-shift of the fluorescence maximum of the photoactive Pchlide-POR-NADPH complexes was observed for a Pchlide:POR ratio between 0.1–0.2, then the maximum was at around 653 nm and the further shift was negligible (Fig. 5).

### Free Pchlide in buffers—a novel method of detection

**Fig 6:**
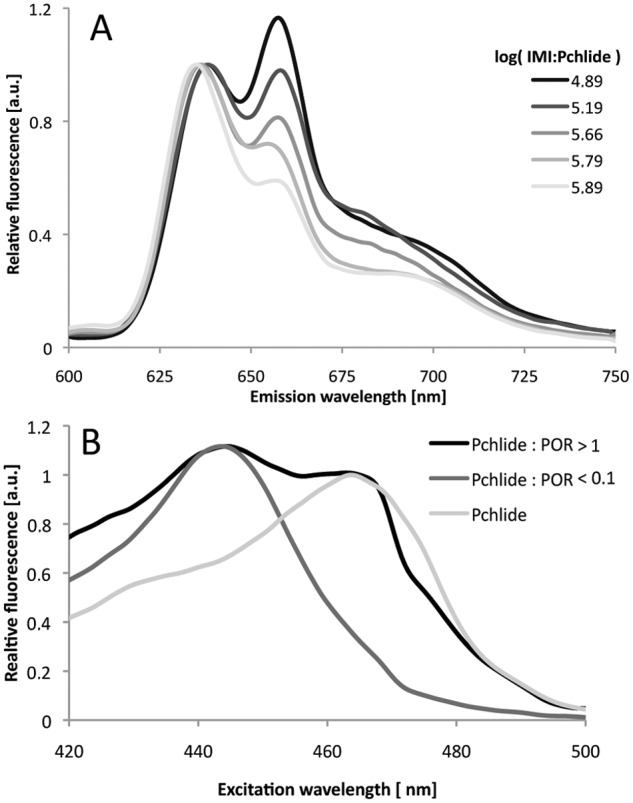
Detection of free Pchlide in buffers. (A) 77 K fluorescence emission spectra of Pchlide in a water-based buffer containing imidazole. Excitation wavelength: 440nm. Pchlide concentration 0.26 μM. (B) 77 K fluorescence excitation spectra for Pchlide in a water-based buffer containing imidazole in the absence and presence of PORA. NADPH concentration: 0.2 mM. Emission wavelength: 660 nm. Pchlide concentration 0.26 μM; log[IMI:Pchlide] = 5.76.

Pchlide unbound to POR active site, which did not undergo photoreduction under the investigated experimental conditions was present in all samples, even for a very low Pchlide:POR ratios. Nevertheless, the fluorescence band of this unbound Pchlide was only present as a shoulder (or asymmetry) hidden within the main fluorescence band of the photoactive Pchlide complexes and became apparent only in the spectra measured after illumination. Looking for a way to detect Pchlide not bound in enzyme-substrate complexes in the reaction mixture before illumination, we took advantage of our observation that the addition of imidazole to Pchlide in water-based buffers resulted in the appearance of an additional emission band with a maximum at 660 nm, which has a characteristic excitation band with a maximum at 465 nm (Fig. 6). These bands probably originated from complexes of Pchlide with imidazole, although detailed analysis of these complexes is beyond the scope of the current paper. The intensities of both excitation and emission bands depended on the relative ratio of imidazole (IMI) and Pchlide concentrations. In particular, for IMI:Pchlide ratios, which corresponded to those in the investigated reaction mixtures, two bands in the fluorescence emission spectrum were observed. These bands had maxima at around 640 and 660 nm for excitation at 440 nm (Fig. 6A). The latter emission band had a characteristic excitation band with a maximum at 465 nm (Fig. 6B).

It was, therefore, possible to observe unbound pigment molecules in reaction mixtures based on fluorescence excitation spectra measured for the emission at 660 nm. The higher the fraction of Pchlide unbound to the enzyme, the higher the intensity of the 465 nm band was observed in the fluorescence excitation spectra (Fig. 6B). The relative intensity of fluorescence originating from the fraction of Pchlide unbound to POR (*F*_*unPchl*_) was calculated according to the following formula:

$$F_{unPch}=\frac{F_{465}-0.2\times F_{440}}{F_{440}+F_{465}}$$ (Eq. 1)

where: F_x_- fluorescence intensity read at x nm from the fluorescence excitation spectrum measured at 77 K for the emission at 660 nm;

The contribution of the band at 440 nm to the intensity at 465 nm in the excitation spectrum (Fig. 6B) was estimated to be 0.2 of the respective intensity at 440 nm. The estimation was done on the basis of the excitation spectrum that was recorded for the sample containing the lowest Pchlide:POR ratio, *i*.*e*. where the 465-nm band was undistinguishable. Obviously, this method provides only semi-quantitive information about the presence of Pchlide molecules unbound to POR and their relative fluorescence intensity. One reason is that differences between the quantum efficiencies of Pchlide-POR-NADPH complexes and the unbound Pchlide were not considered by the formula (Eq. 1). Another is that although some differences in fluorescence maxima were observed for different Pchlide to POR ratios, the excitation spectra were measured for constant emission. In addition, energy migration may also influence the intensities of the different bands in the fluorescence excitation spectra. In spite of that, this method was accurate enough to detect the Pchlide:POR ratio, at which the Pchlide not bound to the enzyme appeared in the reaction mixture.

### POR isozymes and unbound Pchlide

**Fig 7:**
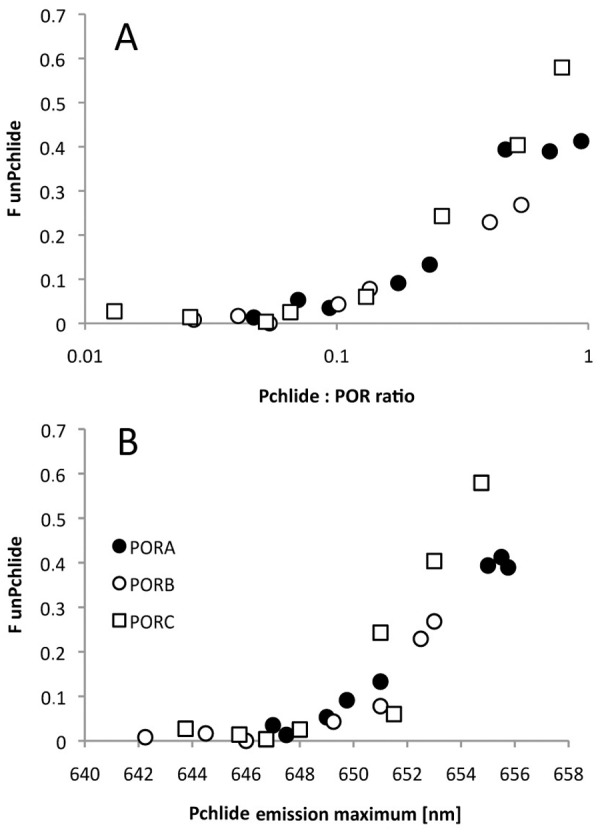
The dependence of the fluorescence of unbound Pchlide (*F*_unPch_) on the Pchlide:POR ratio (A) and on the fluorescence emission maximum (B). *F*_unPch_ was calculated according to formula (Eq. 1) based on the 77 K fluorescence excitation spectra measured for emission at 660 nm. The presented results were obtained from several experiments performed for different POR concentration between 4 and 11 μM, and therefore were analysed with respect to Pchlide:POR ratio.

The method of detection of unbound Pchlide, based on measurements of fluorescence excitation spectra as described above, was then used to detect Pchlide unbound to PORA, PORB and PORC isozymes under the applied experimental conditions. It was observed that an evident 465-nm excitation band measured for emission at 660 nm could be observed already in samples having a Pchlide:POR ratio higher than 0.1 (Fig. 7A). Interestingly, while the band of the unbound Pchlide was absent in samples having an emission maximum shorter than ~ 648 nm, in samples having fluorescence emission maximum above this wavelength its intensity rapidly increased (Fig. 7B).

### Studies of POR activity at room temperature

To compare enzyme activity under more physiological conditions, investigations were performed at room temperature. In this case, the reaction mixture was prepared directly in the fluorescence cuvette in dim green light, and Pchlide was added as the last component. The time of a single measurement was shortened as much as possible, *i*.*e*. the scanning rate was high, to minimize spectral changes in the course of the measurement. The spectra were recorded one after another, thus the fluorometer lamp continuously triggered the Pchlide photoreduction. A representative set of spectra recorded in the course of single experiments is shown in S3 Fig.

**Fig 8:**
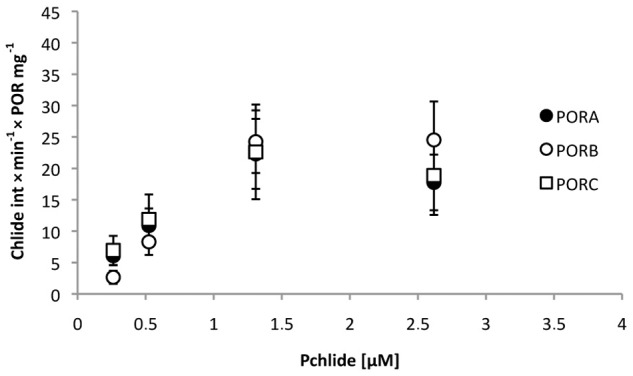
Relative activity of the POR proteins, defined as the increase in Chlide fluorescence intensity per time unit and per protein concentration (in mg), calculated for different Pchlide concentrations. Calculations were performed for series of fluorescence spectra measured at room temperature (*e*.*g*. S2 Fig.). POR concentration in a single experiment was between 1 and 2.75 μM; NADPH concentration: 0.05 mM. Error bars are SD of two independent series of fluorescence spectra.

The fluorescence spectra were composed of two bands, having maxima at around 640–650 nm and 678–690 nm and originating from Pchlide (the substrate) and Chlide (the product), respectively. A decrease in the fluorescence intensity of the former band and an increase of the intensity of the latter was observed in the course of the experiment, indicating the catalytic activity of POR enzymes. Unfortunately, some shifts of the Pchlide maxima of the bands were noticed in the course of illumination, which renders any measurements of the intensity performed at a single point unreliable. The increase in Chlide fluorescence at maximum (*i*.*e*. ~ 680 nm) was analysed as an indicator of the progress of the reaction (see S4 Fig.). Due to the high fluorescence yield of Chlide it appeared as a sensitive indicator of even low pigment concentrations. The relative activity of the POR proteins, defined as the increase in Chlide fluorescence intensity per time unit and per protein concentration showed similar values for PORA, PORB and PORC under these experimental conditions (Fig. 8).

It has to be mentioned that pigment degradation has been observed with illumination of the reaction mixture at room temperature, and for longer than in the present study; this is the subject of ongoing studies.

## Discussion

The homology models of PORA, PORB and PORC, which were developed in the present work, showed a high similarity of structures (Fig. 1A). Nevertheless, spatial orientation of helixes, as well as the shape and alignment of loops, differed among them. This similarity of the PORA, PORB and PORC models seems obvious given the high similarity of their amino acid sequences (Fig. 1B). The identity of *A*. *thaliana* POR sequences was of 79% (*i*.*e*. 73% when including transit peptides), which was the basis of the modelling. In addition, the modelling sessions for all POR isozymes were performed using the same templates for comparison. The present results are quite different from respective models developed by Yuan et al. [22], which showed similarity of spatial structure between *A*. *thaliana* PORA and PORB, and quite different structure for PORC.

It has to be taken into account that homology modelling provides only a static view of protein structure. Under physiological conditions, protein molecules represent dynamic structures and the minor differences that were observed among POR models (Fig. 1A) may be discounted as motions of protein fragments. Nevertheless, based on the analysis of the developed models of PORA, PORB and PORC significant differences in catalytic properties of POR isozymes are not expected, but the existence of some variations in the allosteric regulation of enzyme activity cannot be excluded.

Fluorescence spectroscopy at 77 K has been long used to characterise fluorescence from etiolated seedlings (see for a review [8–9], [23]). Two fluorescence bands were shown to be present in the emission spectra. The first and the main one, originating from Pchlide bound in photoactive complexes with POR and NADPH and having a fluorescence maximum at around 655 nm, can easily be reduced to Chlide even with a pulse of white light. The vibronic satellite of this band was identified at 670 nm [24]. The other fluorescence band, originating from Pchlide unbound to POR and having fluorescence at 633 nm, remains unchanged after illumination. Detailed analysis of the Gaussian components of fluorescence spectra revealed another pool of photoactive Pchlide with emission maxima around 640–645 nm [25–27]. Investigations using circular dichroism have indicated that the more red shifted the maximum of the photoactive Pchlide, the larger the aggregates of Pchlide-POR-NADPH complexes [28]. Analysis of Pchlide properties in model systems showed the ability of this pigment to form aggregates in some organic solvents [17], [29–31], as well as in micelles and liposomes [32]. The aggregates showed red–shifted fluorescence (up to 660 nm) compared to monomers (around 629–642 nm). The large number of papers published so far on Pchlide fluorescence in a variety of natural and artificial systems provides useful reference data for the interpretation of the present results. Pchlide-POR-NADPH complexes reconstituted in this work showed fluorescence maxima between 646 and 656 nm at 77 K (Fig. 2). The red-shift of the maximum followed the increase of the Pchlide:POR ratio in the reaction mixture. Interestingly, the maximum of reconstituted Pchlide-POR-NADPH complexes was similar to that of photoactive Pchlide in PLBs, *i*.*e*. ~ 655 nm [33–34], for a Pchlide:POR ratio higher than 0.2 (Fig. 5). This means that even in the case of Pchlide concentrations significantly lower than that of POR, the applied experimental conditions favour Pchlide-Pchlide interactions observed as the long-wavelength fluorescence band. This might indicate that POR proteins form oligomers in the reaction mixtures which facilitate the interaction of Pchlide bound to adjacent enzyme molecules. It is known that most SDR enzymes are dimers and tetramers and that two long α-helices are involved in oligomerisation [35].

The formation of photoactive Pchlide-POR-NADPH complexes was achieved for PORA, PORB and PORC proteins, and these complexes had similar fluorescence properties dependent only on the Pchlide:POR ratio under the investigated conditions (Fig. 5). The similarity of fluorescence properties, especially at 77 K, seems quite obvious judging from the high similarities of sequences and of the predicted structures of POR proteins (Fig. 1). However, to our knowledge this is the first time such consistent comparison and analysis has been carried out *in vitro*. On the other hand, *A*. *thaliana* POR isoforms operate *in vivo* in plastids having different internal membrane systems and at different developmental stages [15]. Both PORA and PORB accumulate in etioplasts, and have been shown to have a role in PLB formation [15], [36]. In the case of wheat, which has only PORA and PORB, the presence of both proteins in the highly organised PLB structure was confirmed [37–38]. Recently, Yuan et al. [22] have suggested that PLB formation depends on the quantitative level of PORs rather than on the assembly of the photoactive Pchlide-POR-NADPH complexes. PORB is also involved in maintaining Chl biosynthesis throughout the whole plant life and catalyses the reaction in plastids which have developed thylakoid membranes. The expression of the *PORC* gene is upregulated by light and the protein was expressed in light-adapted mature plants [13–14]. Lower Chl production was observed in *porB-1*mutants at very low light intensity, and in *porC-1* mutants at high light intensity as compared to wild-type *Arabidopsis* seedlings grown at the same different light regimes [39]. However, ectopic overexpression of PORA in *porB-1 porC-1* mutant restored normal level of Chl synthesis both at very high and very low light intensities, which indicated that PORA can function over a wide range of ﬂuences [39].

Our results show that the fluorescence red-shift of Pchlide-POR-NADPH complexes, reflecting the oligomerisation of these complexes, is independent of the presence of the plastid lipid lattice. Under the described experimental conditions, the reconstitution of the photoactive Pchlide-POR-NADPH complexes was possible in a homogenous reaction mixture, *i*.*e*. one containing only one POR isozyme and Pchlide *a*, without the addition of any lipids. This is in contrast to the studies of Reinbothe et al. (summarised in [2]) who showed that photoactive complexes having a fluorescence maximum at 660 nm at 77 K can be reconstituted only in a mixture of PORA and PORB of barley, and in the presence of Pchlide *a* and Pchlide *b*. Liposomes were also added to the reaction mixture in that case. Based on those results, a hypothesis about the existence of LHPP (*i*.*e*.‘‘light-harvesting POR:Pchlide”) complexes in the PLBs of etiolated barley was formulated [40], although it was not confirmed by other groups (see for argumentation [1], [41–42]). Recently Yuan et al. [22] suggested that a lack of LHPP complexes in *A*. *thaliana* results from the geometry of POR proteins, which is different for *A*. *thaliana* PORs than that of barley. In the present study all investigated *A*. *thaliana* POR proteins formed photoactive Pchlide-POR-NADPH complexes with long-wavelength fluorescence maxima. It cannot, however, be excluded that the glycerol in the reaction mixture might favour the aggregation process. The influence of glycerol on the red-shift of the fluorescence maxima at 77 K has already been demonstrated for a homogenate of pea epicotyl [43] and for solubilised etioplast membranes [44], although, in both these cases plastid lipids were present in reaction mixtures, which is not the case with the samples analysed in this work.

The recombinant POR proteins of *Synechocystis* [20] (for a review see [45]) and pea [46] have already been investigated. Reconstituted photoactive Pchlide-POR-NADPH complexes had 77 K fluorescence spectra at 644–646 nm. In all these studies, however, detergents (*e*.*g*. Triton X-100) were present in the reaction mixtures, and these may prevent pigment or protein aggregation. It has been shown that treatment of isolated etioplast membranes with detergents leads to disaggregation and finally to degradation of the photoactive complexes in PLBs, and that this was observed as the blue-shift of the fluorescence maxima (*e*.*g*. [34]). In our study, a fluorescence maximum at ~ 646 nm was observed for the lowest investigated Pchlide:POR ratio (*i*.*e*. < 0.1). For such a low relative pigment content, either POR oligomers were strongly unsaturated with Pchlide or only monomers of Pchlide-POR-NADPH were present. In both cases, however, Pchlide-Pchlide interaction had low probability and the observed fluorescence maxima corresponded to those observed in the presence of detergents.

The comparison of the fluorescence spectra recorded before and after illumination confirmed that all the investigated POR isozymes formed photoactive complexes and catalysed Pchlide photoreduction under the applied conditions. However, the Chlide fluorescence maximum was observed in a rather broad range, *i*.*e*. between 677 and 689 nm, and neither correlated with the Pchlide:POR ratio nor depended on the POR isoform. It has already been shown that the position of the fluorescence maximum of newly formed Chlide also provides information about the size of photoactive complexes, and is more red–shifted for more aggregated Chlide:POR:NADP^+^ complexes [25], [47] (for a review see [48]). These researchers [25] observed two Chlide fluorescence bands centred at 684 nm and 694 nm which resulted from the partial photoreduction of Pchlide bound within aggregates of Pchlide-POR-NADPH complexes of different sizes. Therefore, our results indicate either the existence of aggregates of Pchlide-POR-NADPH complexes which are heterogeneous in-size, and/or a rapid release of Chlide from the product-enzyme complexes. The blue-shift of Chlide fluorescence maximum being the result of Chlide release from the product-enzyme complexes for recombinant POR is discussed in [45].

PORC differs from PORA and PORB protein, as far as Chlide production induced by short illumination is concerned. First, the band of Pchlide that remained unreduced after the illumination seemed more complex for PORC than for other POR proteins (Fig. 3). Secondly, relative Chlide fluorescence (Fig. 4) for high Pchlide:POR ratios was evidently lower for PORC than for other POR proteins. Both results indicate lower efficiency of Pchlide photoreduction in the case of PORC and high Pchlide:POR ratios.

Due to the method, presented in this paper, of detecting free Pchlide, which is not bound within pigment-enzyme complexes, it has been shown that the pool of unbound Pchlide appeared already with a low Pchlide:POR ratio (*i*.*e*. ~ 0.1) and its appearance depended on the red-shift of the fluorescence maximum (Fig. 7). This observation points to the existence of POR in the form of oligomers in the reaction mixture and to at least a two-step process of Pchlide binding. Oligomerisation of POR was also confirmed by an analysis of the efficiency of Chlide formation (Fig. 4). For all PORs, this efficiency increases with an increasing Pchlide:POR ratio. The most probable interpretation is that the increasing number of Pchlide molecules bound to POR oligomers observed with an increasing Pchlide:POR ratio favours Pchlide-Pchlide interactions and increases the yield of the photoreaction. At room temperature, all POR proteins converted Pchlide into Chlide, which could be followed by an observation of a decrease in the intensity of the fluorescence band at 640 nm, and an increase in that having a maximum at 684 nm (±6 nm) (S3 Fig.). No differences were observed among POR proteins (Fig. 8). At room temperature, there was no incubation time and the Pchlide bound to POR was immediately reduced to Chlide. POR oligomers may work unsaturated with their substrate. The lack of catalytic differences among POR isoforms at room temperature is in line with the high protein sequence similarity (Fig. 1B).

## Conclusions

We have reconstituted photoactive Pchlide-POR-NADPH complexes of PORA, PORB and PORC proteins, and characterised their steady-state fluorescence properties both at low (77 K) and at room temperature. No differences were found for catalytic activity at room temperature among PORA, PORB and PORC. Prolonged preincubation of the photoactive complexes in the dark revealed that POR probably exists in the form of oligomers in the reaction mixture, and the molecular arrangement and/or environment of pigments may be similar to that observed in PLBs *in vivo*.

We have proved that Pchlide-POR-NADPH complexes showing a long-wavelength fluorescence band can be formed for each of these proteins when supplied with Pchlide *a* only, and without lipid components. Some differences in Pchlide photoconversion efficiency were observed in the case of PORC compared to PORA and PORB.

## Supporting Information

S1 Fig Low temperature (77 K) spectra of Pchlide in control reaction assays measured in darkness (black) and after illumination (grey). Illumination was performed with white light (8 μmol m^-2^ s^-1^ photon flux density). Excitation wavelength: 440 nm. The following control assays are shown: (A) Pchlide (2.6 μM) in WEB buffer. (B) Pchlide (2.6 μM) in WEB buffer with 0.2 mM NADPH. (C) Pchlide (2.6 μM) in WEB buffer with 25% glycerol. (D) Pchlide (2.6 μM) in WEB buffer with PORA (10 μM). (E) Pchlide (2.6 μM) in WEB buffer with 25% glycerol, 150 mM imidazole and PORA (10 μM). (F) Pchlide (2.6 μM) in WEB buffer with PORA (10 μM) and NADPH (0.2 mM).
(TIF)

S2 Fig Low temperature (77 K) fluorescence emission spectra of a reaction mixture containing Pchlide, NADPH and PORC. Spectra labelled as “dark” were measured after 30 min incubation of the reaction mixture in darkness. After these measurements, the samples were thawed, illuminated, frozen again and used for fluorescence measurement (spectra labelled as “light”). “Light 1” and “Light 2” curves represent spectra measured for a 15 sec and 1 min illumination, respectively. See materials and methods for the details. POR concentration: 6.3 ± 0.3 μM, Pchlide: 1.3 μM. Pchlide:POR ratio = 0.21. Excitation wavelength: 440 nm.
(TIF)

S3 Fig Representative fluorescence emission spectra recorded during studies of POR activity at room temperature (see text for details). Excitation wavelength: 440 nm.
(TIF)

S4 Fig Representative results of analysis of the increase of Chlide fluorescence (FChlide) for Pchlide reduction performed at room temperature (see text for details). Each curve shown in the figure represents the time-dependence of the Chlide fluorescence intensity read at the maximum of the band around 680 nm from series of spectra, which example is given in S3 Fig. The rate of Chlide fluorescence increase at time = 0s was calculated for each curve, and showed as a point in Fig. 8. The presented data were obtained for 0.37 mg/ml PORB concentration. NADPH concentration: 0.05 mM in all the experiments.
(TIF)
