# Supplementary figures and images for: Photoactive Protochlorophyllide-Enzyme Complexes Reconstituted with PORA, PORB and PORC Proteins of A. thaliana: Fluorescence and Catalytic Properties
Source: PLoS One. 2015 Feb 6;10(2):e0116990. doi: 10.1371/journal.pone.0116990 (PMC4319759; doi:10.1371/journal.pone.0116990)

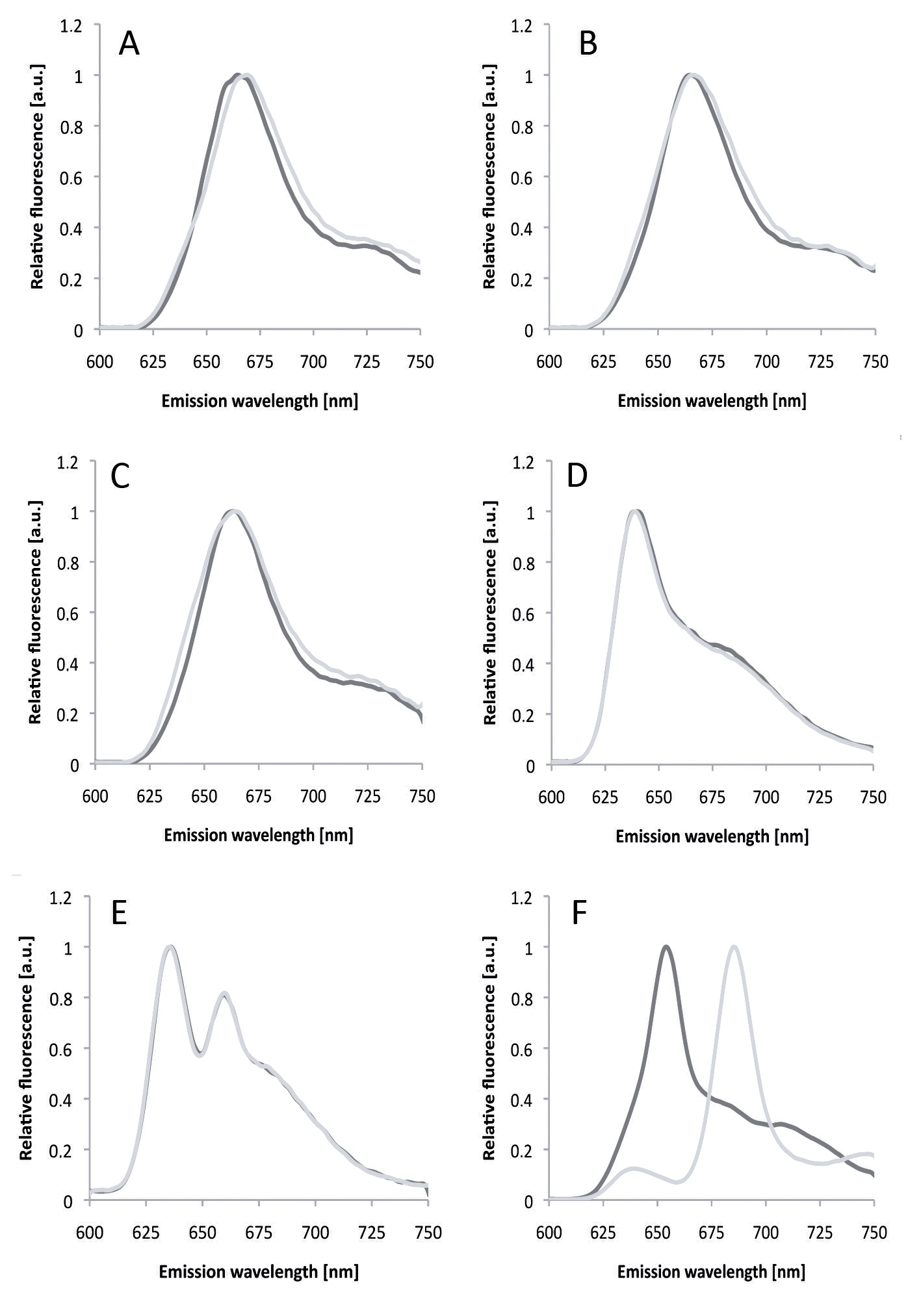

Supplement: S1 Fig — Illumination was performed with white light (8 μmol m-2 s-1 photon flux density). Excitation wavelength: 440 nm. The following control assays are shown: (A) Pchlide (2.6 μM) in WEB buffer. (B) Pchlide (2.6 μM) in WEB buffer with 0.2 mM NADPH. (C) Pchlide (2.6 μM) in WEB buffer with 25% glycerol. (D) Pchlide (2.6 μM) in WEB buffer with PORA (10 μM). (E) Pchlide (2.6 μM) in WEB buffer with 25% glycerol, 150 mM imidazole and PORA (10 μM). (F) Pchlide (2.6 μM) in WEB buffer with PORA (10 μM) and NADPH (0.2 mM). (TIF) [file pone.0116990.s001.tif]

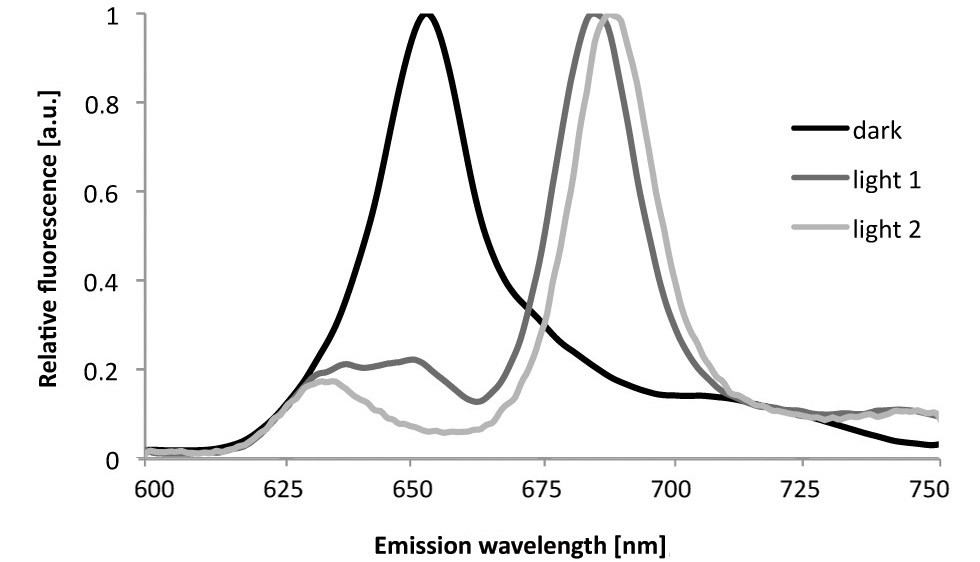

Supplement: S2 Fig — Spectra labelled as “dark” were measured after 30 min incubation of the reaction mixture in darkness. After these measurements, the samples were thawed, illuminated, frozen again and used for fluorescence measurement (spectra labelled as “light”). “Light 1” and “Light 2” curves represent spectra measured for a 15 sec and 1 min illumination, respectively. See materials and methods for the details. POR concentration: 6.3 ± 0.3 μM, Pchlide: 1.3 μM. Pchlide:POR ratio = 0.21. Excitation wavelength: 440 nm. (TIF) [file pone.0116990.s002.tif]

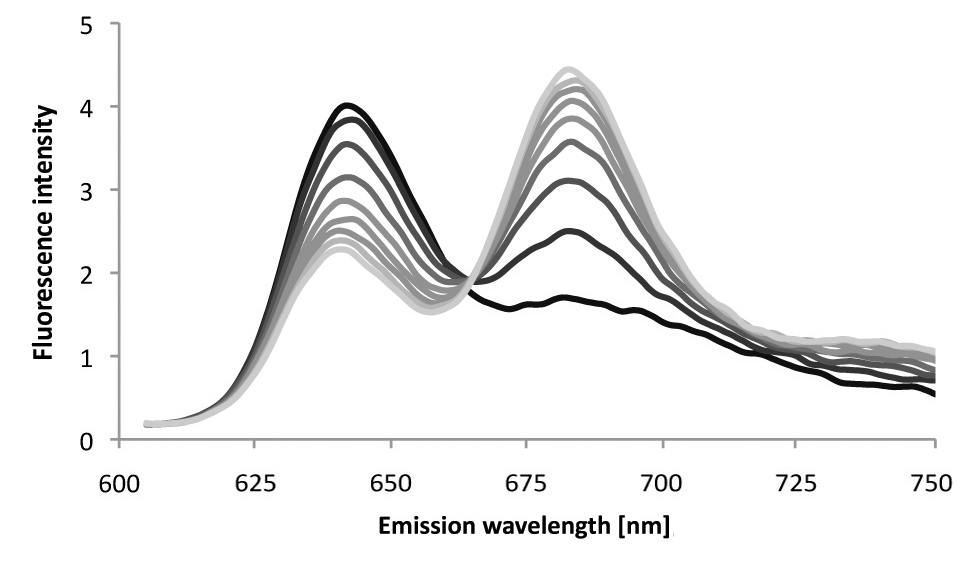

Supplement: S3 Fig — Excitation wavelength: 440 nm. (TIF) [file pone.0116990.s003.tif]

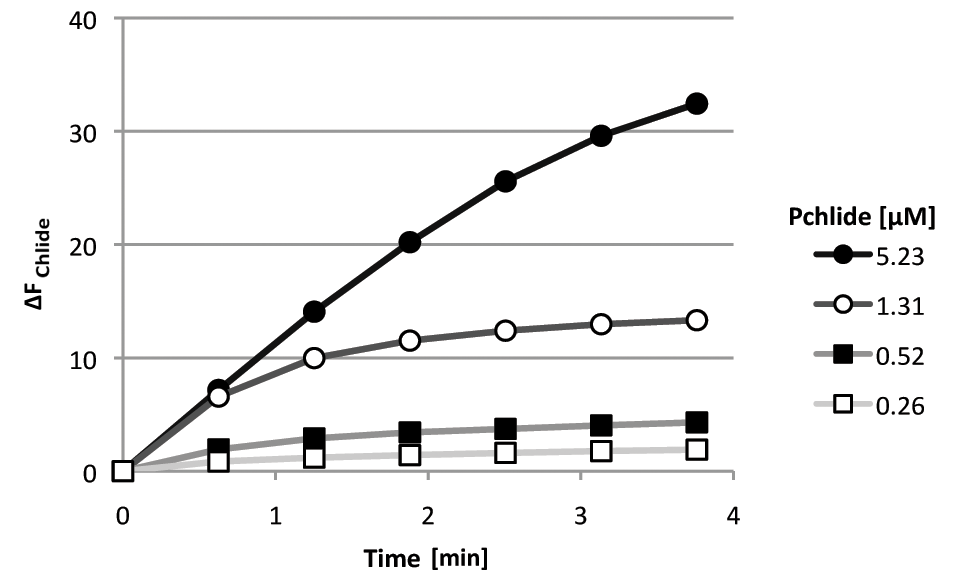

Supplement: S4 Fig — Each curve shown in the figure represents the time-dependence of the Chlide fluorescence intensity read at the maximum of the band around 680 nm from series of spectra, which example is given in S3 Fig. The rate of Chlide fluorescence increase at time = 0s was calculated for each curve, and showed as a point in Fig. 8. The presented data were obtained for 0.37 mg/ml PORB concentration. NADPH concentration: 0.05 mM in all the experiments. (TIF) [file pone.0116990.s004.tif]
